# 基于季铵化烯丙基缩水甘油醚改性的阴离子交换固定相的制备

**DOI:** 10.3724/SP.J.1123.2022.03008

**Published:** 2022-08-08

**Authors:** Zongying LI, Xin CHEN, Feifang ZHANG, Bingcheng YANG

**Affiliations:** 华东理工大学药学院, 上海 200237; School of Pharmacy, East China University of Science and Technology, Shanghai 200237, China

**Keywords:** 离子色谱, 聚甲基丙烯酸缩水甘油酯-二乙烯基苯, 残留双键, 固定相, ion chromatography (IC), poly(glycidylmethacrylate-divinylbenzene) (GMA-DVB), pedant double bonds, stationary phase

## Abstract

制备了一种季铵化烯丙基缩水甘油醚(allyl glycidyl ether, AGE)改性聚合物基质的阴离子固定相应用于离子色谱系统。它是利用AGE与水解的聚甲基丙烯酸缩水甘油酯-二乙烯基苯(poly(glycidylmethacrylate-divinylbenzene, GMA-DVB)微球表面残留双键通过表面自由基共聚,再通过*N*,*N*-二甲基乙醇胺(一种叔胺)进行开环反应制备得到的。通过考察有机叔胺类型、微球水解、单体和引发剂用量、反应温度和时间对7种阴离子分离性能的影响,优化了制备条件。采用扫描电镜、元素分析对所得阴离子固定相进行了表征。结果表明,采用预先水解的GMA-DVB微球(水解过程中微球表面丰富的环氧基团转化为羟基)相对于直接采用GMA-DVB微球有助于降低固定相的交换容量和微球自身的非离子吸附作用;通过淋洗液浓度和目标离子保留因子的拟合结果证实了该固定相保留机理为典型的离子交换作用。使用碳酸根淋洗液,在优化的色谱条件下,该固定相可在13 min内实现常见7种无机阴离子的基线分离,并表现出较高的柱效(Cl^-^理论塔板数为49000块/m)。该色谱柱实用性通过分析自来水实际样品进行了验证。

## Abstract (en)

A polymer-based strong anion stationary phase modified by quaternary ammoniated allyl glycidyl ether (AGE) for ion chromatography (IC) was developed. It was prepared by surface copolymerization between AGE and the pedant double bonds associated with hydrolyzed poly(glycidylmethacrylate-divinylbenzene) (GMA-DVB) substrate, followed by quaternization with *N*,*N*-methyldiethanolamine (a tertiary amine, MDEA). The synthesis conditions were optimized, including the type of organic tertiary amines (MDEA, *N*,*N*-dimethylethanolamine, trimethylamine), substrate hydrolysis, the amount of monomer and initiator, reaction temperature and reaction time. The obtained anion stationary phase was characterized by scanning electron microscope and elemental analysis. MDEA was observed to be the best quaternization reagent since the anion exchanger obtained by such reagent showed good separation and suitable retention time towards model inorganic anions. The resulting reason probably lies in higher hydrophilicity of MDEA relative the other two ones. The use of rich epoxy groups of GMA-DVB to introduce the functional groups was commonly used in many previous reports. Here the epoxy groups were firstly hydrolyzed to be diol groups and the pendant double bonds onto the surface of GMA-DVB particles were used to graft AGE. The data achieved indicated that the use of hydrolyzed GMA-DVB substrate would be helpful to reduce anion exchange capacity and unwanted non-ion exchange interaction, in which the rich epoxy groups were converted to hydroxy groups. More important, the obtained anion exchanger after hydrolytic treatment could offer a significantly reduction of retention time (~68%) and a ~1.67-fold higher plate count (take Br^-^ as an example). The apparent capacity of the phase was computed to be 264 μmol/g by content of N data and its effective capacity was measured to be 98.5 μmol/column by the breakthrough curve method. Under the optimal chromatographic conditions, the obtained stationary phase showed baseline separation of seven common inorganic anions in less than 13 min using carbonate-bicarbonate mixed eluent, exhibiting high separation efficiency and peak shape, e. g. 49000 plate/m and 38000 plate/m of the theoretical plate counts respectively for chloride and nitrate, and their asymmetric factor were 1.3 and 1.4. These can be comparable or slightly higher than those of commercial columns. The fitted equation of the eluent concentration and the retention factors of model anions was proved that ion exchange model dominates the retention mechanism of the anion stationary phase. This offers a simple way to prepare anion exchanger and to manipulate anion exchange capacity. The utility of the obtained anion exchanger has been demonstrated to the analysis of tap water.

离子色谱(ion chromatography, IC)是分析无机阴离子最常用的技术。新型阴离子固定相的开发一直是IC领域的研究热点^[1]^。聚合物基质固定相由于pH耐受范围宽和化学稳定性高等优点,在IC系统中占据着绝大部分应用。目前常用的聚合物基质多为苯乙烯-二乙烯基苯(polystyrene-divinylbenzene, PS-DVB)、乙基乙烯基苯-二乙烯基苯、甲基丙烯酸酯^[2]^。近年来,聚甲基丙烯酸缩水甘油酯-二乙烯基苯基(poly(glycidylmethacrylate-divinylbenzene, GMA-DVB)固定相因其表面含有环氧基团和残留双键,易于进行表面改性而备受关注^[3⇓⇓-6]^。Liu等^[7]^利用GMA-DVB表面的环氧基团,通过二环氧化物和甲胺的超支化反应,制备得到阴离子固定相。该固定相具有极高的交换容量,分析常规阴离子因保留过强而不具有优势,但对弱保留组分比如有机弱酸表现出良好的分离效果;与DVB相关的残留双键已被证明具有活性^[8]^,可以在引发剂作用下与含不饱和键的功能单体接枝得到阴离子固定相。Kaltz等^[9]^通过将自行合成的功能单体与残留双键共聚得到一种阴离子固定相,并探索了聚合机理。通过直接接枝甲基丙烯酸二甲氨基乙酯甲基氯化物(dimethylaminoethyl methacrylate methylchloride, DMC),作者所在实验室报道了一种阴离子固定相,对常见7种无机阴离子表现出良好的分离效果^[10]^。该方法具有合成步骤简单、重复性好等优点,其不足在于固定相交换容量较高,需要高浓度的淋洗液,这就使得抑制背景电导噪声偏大。实际上,许多基于GMA-DVB的阴离子固定相中都有类似的缺陷^[4]^;另外,DMC尚无高纯度商品化试剂,会导致副反应发生,更重要的是,DMC结构中所含酯基在碱性溶液中会缓慢水解。考虑到有市售高纯度(纯度>99%)烯丙基缩水甘油醚(allyl glycidyl ether, AGE),其分子结构中所含的醚基比DMC的酯基理论上更稳定,本文拟通过AGE代替DMC,通过自由基反应键合到水解的GMA-DVB微球表面,再用合适的叔胺铵化得到最终的阴离子固定相,以期得到合适交换容量的固定相,同时降低来自基球本身的非离子性作用。

## 1 实验部分

### 1.1 仪器

数显控温油浴锅(德国IKA公司); KH-100E超声波清洗器(宁波新芝生物科技有限公司); TriStar Ⅱ 3020多通道全自动比表面积分析仪(美国Micromeritics公司); Varil EL Ⅲ元素分析仪(德国Elementar公司);离子色谱仪IC-2010(日本TOSOH公司); KOH淋洗液发生器(EDG-100)、电致膜抑制器(AES-100)(苏州明昊色谱技术有限公司)。

### 1.2 试剂与材料

AGE(纯度99%)、偶氮二异丁腈(AIBN)、邻苯二甲酸二丁酯(DBP,纯度99%)、*N*,*N*-甲基二乙醇胺(MDEA,纯度99%)、*N*,*N*-二甲基乙醇胺(DMEA,纯度99%)、过氧化苯甲酰(benzoyl peroxide, BPO)、聚乙烯醇(PVA)、三甲胺水溶液(TMA)均购自上海阿拉丁公司;十二烷基硫酸钠(SDS)购自上海聚源公司。其他试剂均为分析纯,购自上海凌峰公司。除非另有说明,溶液均采用电阻率18.2 MΩ·cm的超纯水(美国Millipore)配制。

### 1.3 阴离子色谱固定相的合成

GMA-DVB微球按照之前报道^[10]^合成得到。简述如下:称取聚苯乙烯种子1.0 g,加入0.2% SDS水溶液20 mL,缓慢搅拌均匀;另取8 mL DBP,加入40 mL 0.2% SDS水溶液,超声乳化后与上述种子溶液合并,在30 ℃下活化24 h得到活化的种子溶液;将含有10 mL GMA、22 mL DVB、28 mL甲苯、0.4 g BPO、1.6 g SDS的2% PVA水溶液300 mL超声乳化均匀后加入到活化种子溶液中。在30 ℃进行溶胀,保持24 h。然后通氮气除氧,溶液温度升温到70 ℃,继续反应24 h。待反应结束后,抽滤,用水洗涤至无泡沫产生,随后用乙醇、二氯甲烷充分洗涤,抽滤后50 ℃干燥即可得到GMA-DVB微球。

该微球首先进行水解处理:在65 ℃下浸入稀硫酸水溶液(0.1 mol/L)中2 h;过滤后残留物用水洗至中性,60 ℃干燥过夜,所得填料命名为GH。将2.5 g GH、0.42 g AIBN和0.2 g SDS放入装有90 mL乙醇的250 mL烧瓶中。对混合物进行超声处理至颗粒分散均匀。加入7.5 mL(63 mmol)AGE,在氮气保护下70 ℃反应6 h。过滤后,残渣依次用乙醇和水洗涤,命名为GH-AGE;加入10.6 mL (94.5 mmol)MDEA,与GH-AGE在65 ℃反应6 h。过滤后,残留物用水洗涤并在60 ℃干燥12 h,得到最终的阴离子固定相,命名为GH-AGE-MDEA。其合成路线如图1所示。

**图1:**
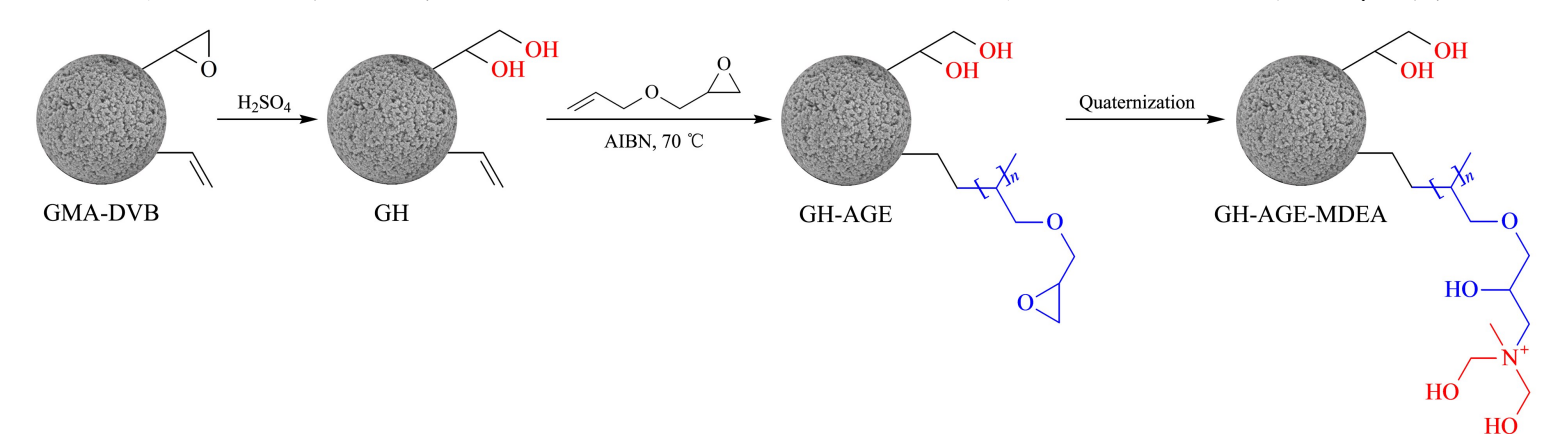
GH-AGE-MDEA阴离子交换固定相合成路线示意图

为了比较,制备了一种未经水解处理的阴离子固定相,命名为G-AGE-MDEA。

将2.0 g GH-AGE-MDEA和20 mL纯水以浆料形式填充到不锈钢柱(150 mm×4.6 mm)中,以纯水作为匀浆液和顶替液,填充压力为35 MPa。使用前将得到的色谱柱用碳酸钾溶液冲洗至少12 h。

### 1.4 色谱柱交换容量的测定

色谱柱交换容量采用突破曲线法测定^[11]^:先用50 mmol/L NaCl水溶液以0.5 mL/min的流速冲洗4 h,再用去离子水以0.5 mL/min的流速冲洗2 h。最后用5 mmol/L NaNO_3_水溶液冲洗,其流出物通过紫外检测器监控,检测波长为210 nm,以 1 mL/min流速冲洗色谱柱直至突破。离子交换容量*Q*(μmol/column)按下式计算:

*Q=C×F×*(*t*_b_-*t*_0_)

式中*C*为NaNO_3_浓度(mmol/L); *F*为NaNO_3_溶液流速(mL/min); *t*_b_为突破时间(min); *t*_0_为死时间(min)。

### 1.5 色谱条件

色谱柱:实验室自制阴离子交换固定相GH-AGE-MDEA(150 mm×4.6 mm, 5.7 μm);流动相:2.0 mmol/L K_2_CO_3_+2.5 mmol/L KHCO_3_;进样量:30 μL;流速:1.0 mL/min;抑制器电流:17 mA;柱温:35 ℃。

## 2 结果与讨论

### 2.1 固定相的表征

GMA-DVB微球和GH-AGE-MDEA的扫描电子显微照片如图2a所示。可以看出两种微球均表现出良好的单分散性,平均粒径分别为5.5 μm和5.7 μm。二者粒径差别很小。这表明在合成过程中GMA-DVB单分散性保持良好;图2b和图2c分别是两种微球的孔径分布曲线和氮气吸附-脱附曲线。可以看出,40~60 nm的微孔在GH-AGE-MDEA结构中占主导;此外,由两种微球的元素分析数据(见表1)可以看出,相对于GMA-DVB氮含量0.16%, GH-AGE-MDEA的氮含量(0.53%)明显增加。理论上,GMA-DVB氮含量为零,少量的氮含量可能是由于空气中氮的干扰或聚合过程中AIBN的残留造成的,之前报道也观察到类似的现象^[10,12]^。GH-AGE-MDEA的理论和实际交换容量分别为264 μmol/g和98.5 μmol/column。这些表征说明AGE已成功键合到GMA-DVB微球表面。

**图2:**
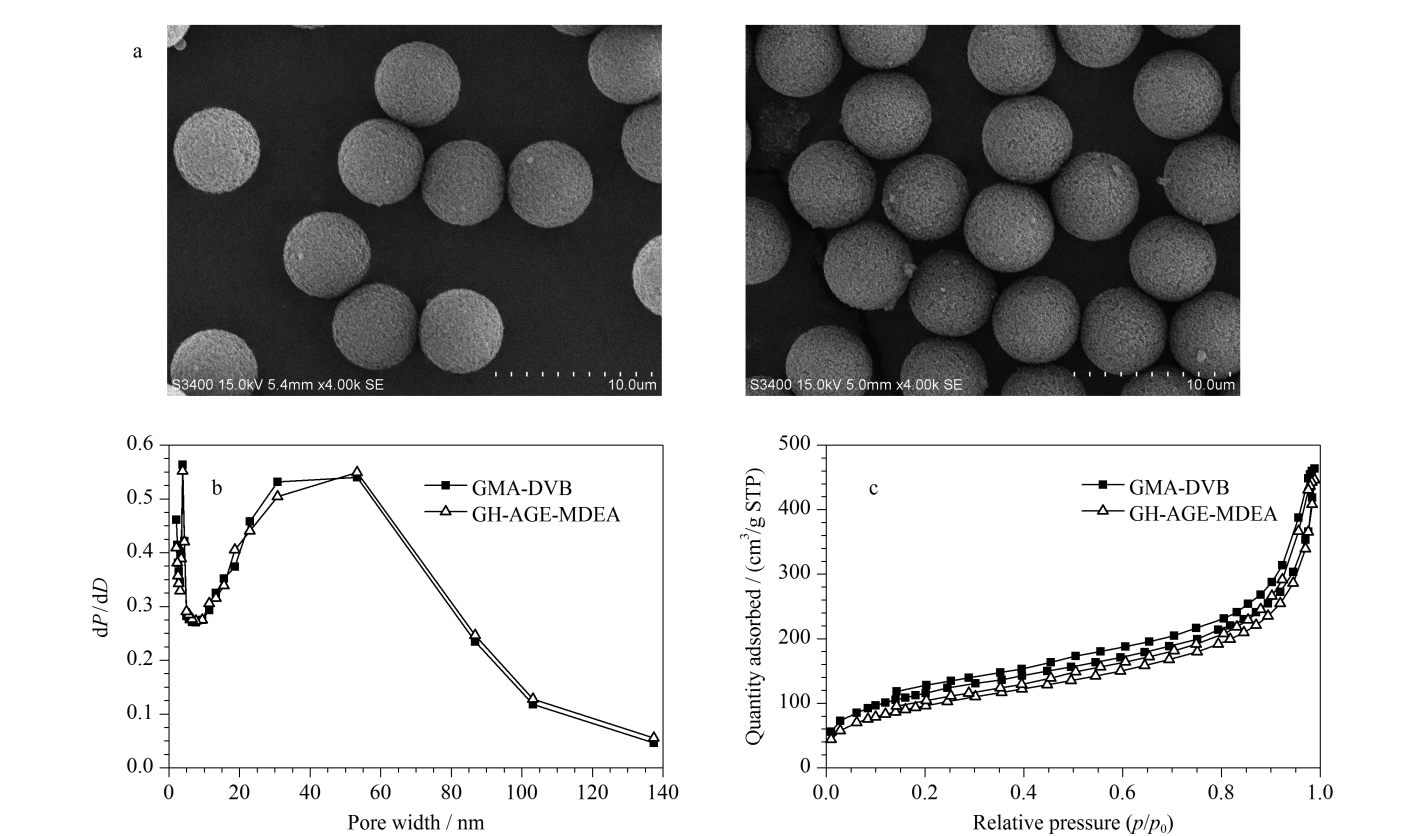
GMA-DVB和GH-AGE-MDEA的(a)SEM图、(b)孔径分布和(c)BJH-吸附脱附曲线

**表1:** GMA-DVB和GH-AGE-MDEA的元素分析结果

Stationary phase	C/%	H/%	N/%
GMA-DVB	79.18	7.57	0.16
GH-AGE-MDEA	74.13	7.68	0.53

### 2.2 合成条件的优化

#### 2.2.1 季铵化试剂的选择

阴离子固定相的保留选择性主要受离子交换位点,离子交换位点周围取代基以及基质共同影响^[13]^。为探讨离子交换位点及取代物的影响,尝试了3种叔胺作为开环试剂,包括MDEA、DMEA和TMA。所得固定相分别命名为GH-AGE-MDEA、GH-AGE-DMEA和GH-AGE-TMA。如图3a所示,3种固定相对阴离子的保留有显著差异。后两种在合理时间内无法观察到7种阴离子的全部洗脱,在30 min内仅有F^-^、Cl^-^被洗脱,且Cl^-^峰表现出明显的展宽;相比之下,GH-AGE-MDEA可以在<20 min内实现7种阴离子的基线分离。3种固定相的离子交换位点均为季氨基,出现上述这种现象理应是离子交换位点周围取代基不同所致。3种有机胺亲水性顺序依次为MDEA>DMEA>TMA,相应获得的阴离子固定相的亲水性顺序等同。GH-AGE-MDEA中所含季铵离子交换位点相邻两个*β*-羟基使其水合度增强,有利于高度水合的C $O_{3}^{2-}$ 和OH^-^靠近季氨基,从而有助于增强洗脱能力。该现象与之前文献^[14]^报道比较相近;其他原因还包括交换容量不同。3种季铵化试剂的化学结构不同,由于空间位阻不同,会影响开环反应中的接枝量,进而导致阴离子固定相的交换容量不同。这可以通过测量柱容量得到证实:3者所得固定相交换容量分别为98.5、230.2、242.5 μmol/column。GH-AGE-MDEA交换容量明显低于其他两种,可以在合理时间内实现对目标离子的分离。因此,MDEA被选为最优开环试剂。

**图3:**
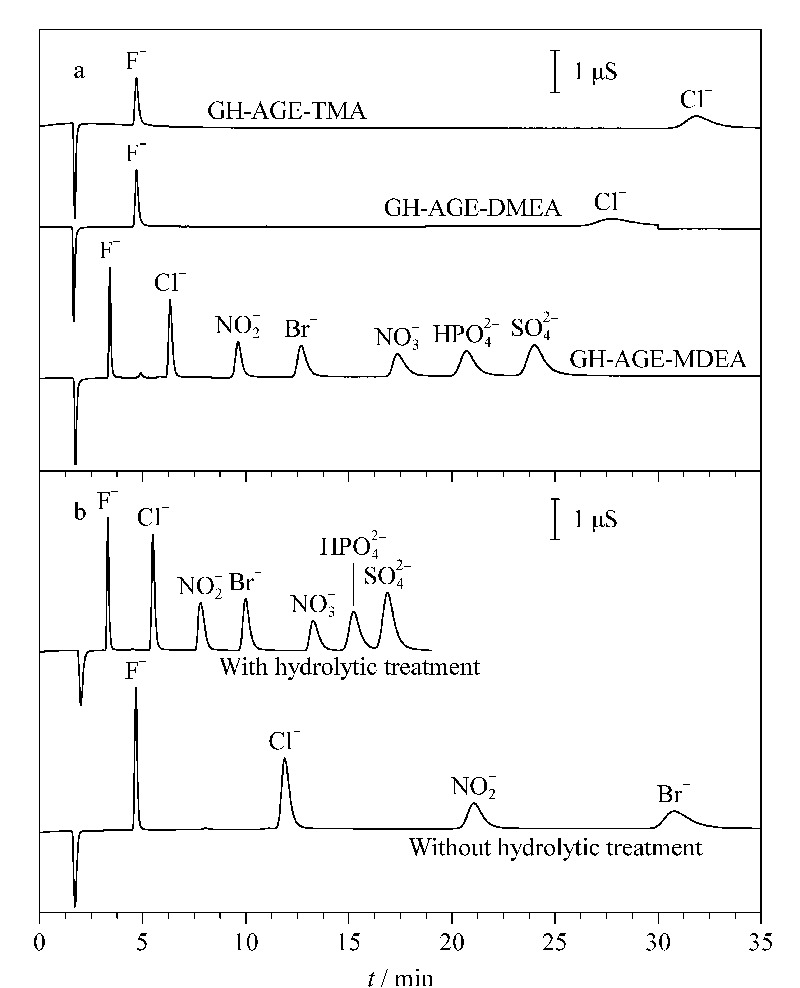
(a)阴离子固定相和(b)水解作用对7种阴离子分离的影响

#### 2.2.2 GMA-DVB基球水解处理的影响

阴离子固定相实际分离效果还受基质自身的影响。为消除基质可能的影响,本文的策略是通过水解使与GMA相关的环氧基团失活变成双醇基。一则确保它们不参与季铵化反应,避免之前报道中交换容量过高的弊端^[7]^,同时还引入了高亲水的双醇基,有助于降低基质的吸附作用。如图3b所示,未经水解处理的模型离子在G-AGE-MDEA上的保留非常强,比如N $O_{3}^{-}$ 无法在50 min内洗脱,二价阴离子(例如S $O_{4}^{2-}$ )在80 min内不能被洗脱。相比之下,水解后所得固定相保留时间会显著下降,7种离子可以在20 min内被洗脱,同时伴随着柱效的增强。以Br^-^为例,其保留时间减少了68%,理论塔板数提高了1.67倍。究其原因,很大程度上应该是来自于水解处理后柱交换容量的降低,基质吸附作用的下降亦有一定贡献。因此,在后续制备阴离子固定相过程中均采用水解后基球作为起始原料。

#### 2.2.3 单体量和时间对分离的影响

AGE接枝过程属于自由基聚合,单体用量、反应温度和时间都会影响最终的交换容量。图4a所示是不同单体用量制备得到的阴离子固定相对分离的影响。当AGE用量为42 mmol时,除硝酸根和磷酸氢根共洗脱外,其他5种阴离子分离良好;当AGE量增加到63 mmol时,7种离子均达到基线分离;进一步增加到84 mmol,几种离子的分离效果得到进一步改善,但保留时间也相应增加,峰高和塔板数也随之下降。因此,单体用量经优化后选择为63 mmol。

**图4:**
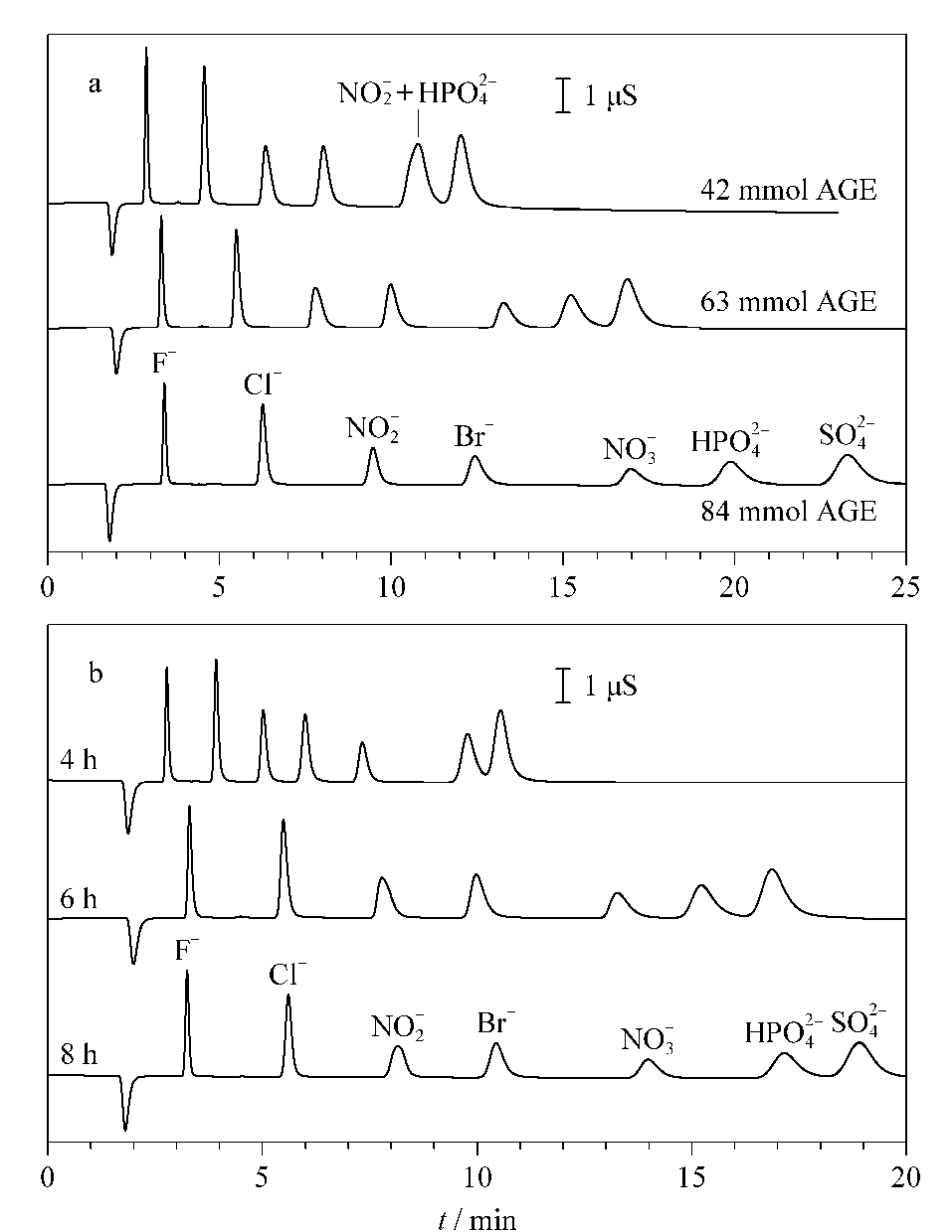
(a)单体AGE用量和(b)反应时间对分离的影响

控制反应温度为70 ℃时,探讨了反应时间的影响,如图4b。反应时间为4~8 h时,随着反应时间的增长,目标离子的保留时间逐渐增加,分离度改善,但理论塔板数也会随之下降。综合考虑,选择6 h为最佳反应时间。

### 2.3 色谱性能

所制备的色谱柱分离常见无机阴离子的典型色谱图如图5所示。7种无机阴离子可在13 min内实现良好的分离,且分离效率高、峰形良好。以Cl^-^和N $O_{3}^{-}$ 为例,其理论塔板数分别为49000块/m和38000块/m;其不对称因子分别为1.3和1.4。由于基质粒径不同,不同色谱柱之间很难直接比较柱效。本文根据文献报道,进行了一个大致的对比:以Cl^-^为例,IonPac^TM^ AS22-Fast色谱柱的理论塔板数为34150块/m^[15]^;文献[10]和文献[16]中色谱柱给出的理论塔板数分别为25600块/m和34000块/m。简单的对比可以看出本文所报道色谱柱具有良好的分离效率。

**图5:**
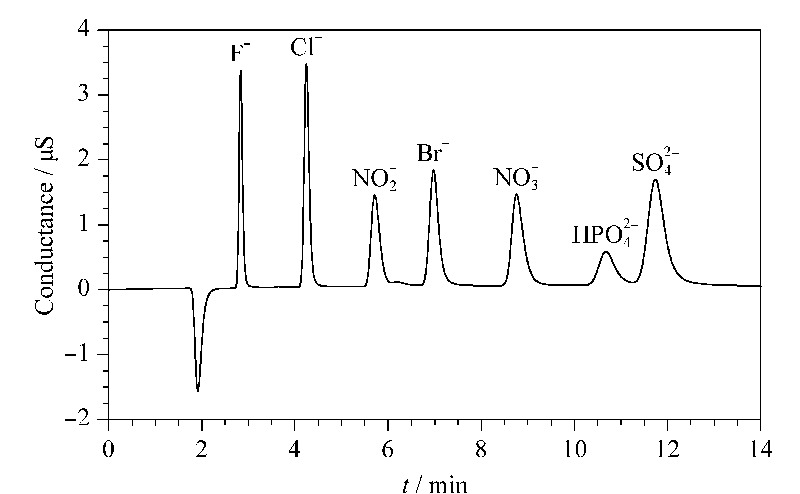
GH-AGE-MDEA阴离子交换固定相分离7种 阴离子的色谱图

用Cl^-^、N $O_{3}^{-}$ 、S $O_{4}^{2-}$ 3种离子考察G-AGE-MDEA的保留机理。保留因子*k*与碳酸盐浓度*C*的对数呈良好的线性相关。3种离子拟合曲线的斜率分别为-0.561、-0.506和-1.172,与理论斜率-0.5(1价离子)或-1.0(2价离子)非常吻合。这表明该固定相保留机理为典型的离子交换作用。

色谱柱运行稳定性通过考察目标离子保留时间的日内和日间相对标准偏差(RSD),分别为0.09%和1.42%;使用2 mmol/L K_2_CO_3_+2.5 mmol/L KHCO_3_洗脱液连续冲洗超过1500倍柱体积,目标离子的保留时间和峰面积RSD分别小于2.7%和1.8%,这显示出该色谱柱良好的稳定性。

GH-AGE-MDEA阴离子交换固定相的一个典型应用是自来水分析。在5~250 μmol/L范围内得到7种常见阴离子的外标校准曲线,相关系数*R*^2^均>0.9994。自来水中Cl^-^、N $O_{3}^{-}$ 、S $O_{4}^{2-}$ 的测定浓度分别为27.12、104.5和29.57 μmol/L。

## 3 结论

本文报道了一种聚合物基质阴离子固定相。它是通过将AGE接枝到水解GMA-DVB微球上,再季铵化处理。DVB的残留双键活性足够引入一定数量的AGE,同时将GMA游离环氧基团水解为双醇基,可以得到交换容量适中、基质干扰作用小的阴离子固定相。
